# A new species of the genus *Hypocharassus* Mik (Diptera, Dolichopodidae) from Korea

**DOI:** 10.3897/BDJ.10.e82851

**Published:** 2022-06-08

**Authors:** Young-Kun Kim, Sang Jae Suh

**Affiliations:** 1 Kyungpook National University, Daegu, Republic of Korea Kyungpook National University Daegu Republic of Korea

**Keywords:** new species, Korea, Dolichopodidae, Hydrophorinae, Hypocharassini, Hypocharassus

## Abstract

**Background:**

The genus *Hypocharassus* Mik, 1879 has only been recorded in the Nearctic and Oriental regions and, to date, it contains four known species.

**New information:**

*Hypocharassuscavitarsus* **sp. n.** is described from Korea. This is the first record of this genus in the Palearctic Region. A description of the new species and a key to the *Hypocharassus* species are presented herein.

## Introduction

The genus *Hypocharassus* Mik, 1879 contains four species and has only been recorded in the Nearctic and the Oriental Regions. This genus was erected by Mik (1878), who identified a new species, *H.gladiator*, from Georgia, USA. Similarly, Wheeler (1898) established a new genus, *Drepanomyia*, based on *D.pruinosus* (= *H.pruinosus*) from Florida, USA; however, Coquillett (1910) considered this genus a junior synonym of *Hypocharassus*. Later, *H.farinosus* and *H.sinensis* were recorded from Taiwan and Guangxi, China, by Becker (1922) and Yang (1998), respectively.

Adult flies of this genus can be found on wet seashore sand or among the grass of the sea meadow. They usually fly close to the ground within a height of approximately 1 m and are not easily captured by simply swinging around the catching net (Wheeler 1900, Smith 1952). Their egg-shaped cocoons, which are composed of sand and adhesive material, are easily found on the beach sand, but their habitats and the feeding behavior of larvae are not well known. Previous reports suggested that the larvae live in the spaces between sand grains and eat small Crustacea or marine plankton (Smith 1952).

This study describes *Hypocharassuscavitarsus* sp. n. as a new species from the southern coast of Korea and reports the genus *Hypocharassus* in the Palearctic Region for the first time.

## Materials and methods

Morphological features were photographed using a stereomicroscope (Olympus SZX 16; Olympus, Tokyo, Japan), compound microscope (Olympus BX50) and Michrome 16 CMOS camera (Tucsen, China). Specimens are preserved in dry condition for observing and 95% ethanol for later molecular diagnosis. All specimens examined in this study were deposited in the collection of the School of Applied Biosciences, Kyungpook National University, Daegu, Korea.

## Taxon treatments

### Hypocharassus cavitarsus sp. n.

6E65F3BD-006E-5968-A9DD-E929EBE55829

9878BD39-6122-42D8-AA69-F29094F1A581

#### Materials

- **Type status:** Holotype. **Occurrence:** recordedBy: Young-Kun Kim & Sang Jae Suh; sex: male; lifeStage: adult; **Taxon:** scientificName: *Hypocharassuscavitarsus*; family: Dolichopodidae; **Location:** country: Korea; countryCode: KR; stateProvince: Jeollanam-do; county: Sinan-gun; municipality: Imja-myeon; locality: Samdu-ri; verbatimLatitude: 35°03'46.7"N; verbatimLongitude: 126°03'31.2"E; **Event:** year: 2021; month: 6; day: 19
- **Type status:** Paratype. **Occurrence:** recordedBy: Young-Kun Kim & Sang Jae Suh; sex: 4 females; lifeStage: adult; **Taxon:** scientificName: *Hypocharassuscavitarsus*; family: Dolichopodidae; **Location:** country: Korea; countryCode: KR; stateProvince: Jeollanam-do; county: Sinan-gun; municipality: Imja-myeon; locality: Samdu-ri; verbatimLatitude: 35°03'46.7"N; verbatimLongitude: 126°03'31.2"E; **Event:** year: 2021; month: 6; day: 19
- **Type status:** Paratype. **Occurrence:** recordedBy: Young-Kun Kim & Sang Jae Suh; sex: 11 males,7 females; lifeStage: adult; **Taxon:** scientificName: *Hypocharassuscavitarsus*; family: Dolichopodidae; **Location:** country: Korea; countryCode: KR; stateProvince: Chungcheongnam-do; county: Taean-gun; municipality: Taean-eup; locality: Donae-ri; verbatimLatitude: 36°49'03.4"N; verbatimLongitude: 126°19'17.9"E; **Event:** year: 2021; month: 7; day: 14

#### Description

**Figure 1.**
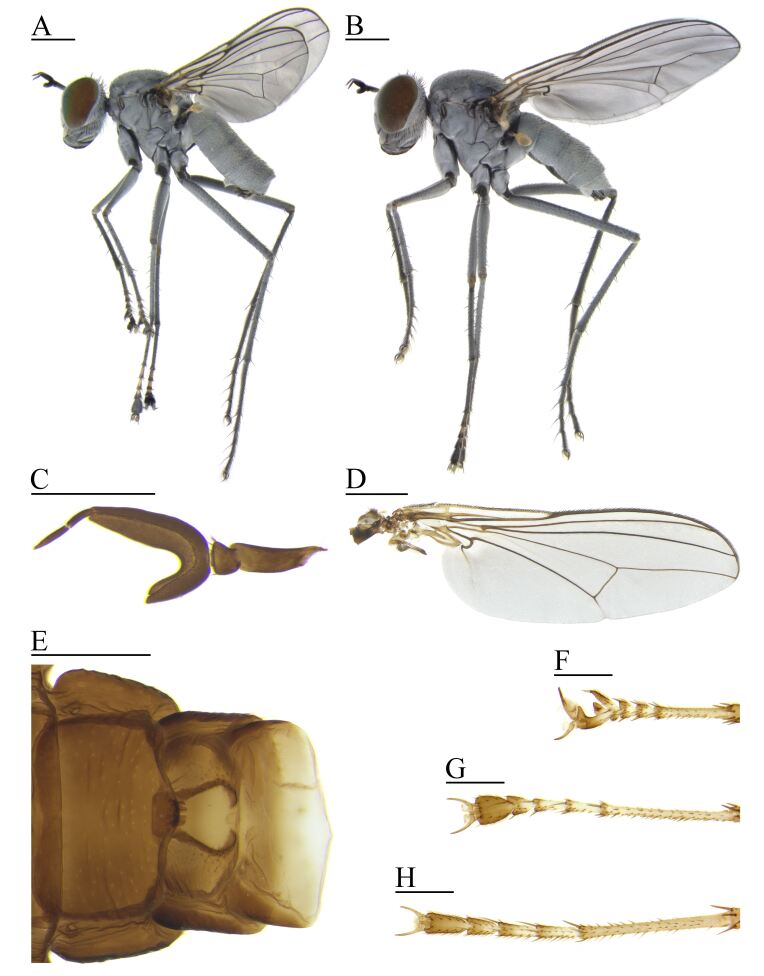
*Hypocharassuscavitarsus* sp. n. **A** male, lateral view; **B** female, lateral view; **C** male antenna, lateral view; **D** male wing, lateral view; **E** male fourth and fifth sternite, ventral view; **F** male fore tarsus, dorsal view; **G** male mid tarsus, dorsal view; **H** male hind tarsus, dorsal view. Scale bars: A-B and D = 1 mm; C, E-H = 0.5 mm.

***Male*** (Fig. 1A)

**Head** inverted triangle-shape at anterior view and metallic bluish green with thick whitish pruinosity; vertex slightly concave; ocellar triangle convex; 3 ocellus present; ocellar seta almost straight and divergent and as long as distance between median ocellus and lateral ocellus; postoceller almost straight and approximately 0.5 times shorter than ocellar seta; vertical seta proclinate and approximately 0.5 times shorter than ocellar seta (rarely absent in some specimens); postvertical seta proclinate and convergent and approximately 1–2 times longer than ocellar seta; frons broadened upwards; width between frons and face as long as compound eye at anterior view; face slightly broadened downwards; clypeus subparallel downwards and apically round; compound eye with tiny pale setulae between facets; upper postocular setae black in a single row; lower postocular setae pale and combined with occipital setae; upper occiput flat; lower occiput with thick pale setae; antenna black; scape bare; pedicel setose at apical margin; postpedicel check mark shape and ventral projection 2/5 times shorter than dorsal projection; arista-like stylus thick and two segmented and apical segment minutely longer than basal segment and placed apically; palpus basally grey and apically yellow with pale setulae; proboscis large and black with pale setulae (Fig. 1C).

**Thorax** metallic bluish green with thick whitish pruinosity and mainly black setae; mesonotum with violet vittae between acrostichal and dorsocental setae starting from anterior scutum and then fading before reaching the scutellum; 3–16 tiny acrostichal setae irregularly biseriate; 14–18 dorsocentral setae tiny, except the long last one; postpronotal lobe with 1 (rarely 2) seta and some pale setulae; 3–5 tiny intra-alar setae, 1 (rarely 2) presutural supra-alar, 3 postsutural supra-alar, 1 (rarely 2) notopleural, 1 postalar setae present; posterial scutum round; scutellum approximately 0.5 times shorter than width and apically round; 2–3 (rarely 4) scutellar setae present and as long as length of scutellum and laterals slightly smaller than median seta; proepisternum with pale setulae; anepisternum, katepisternum, anepimeron, katepimeron, meron and laterotergite bare.

**Legs** mainly metallic bluish green with thick whitish pruinosity; all coxae and trochanter with pale setulae; fore femur and mid femur with pale setulae and 1 apical posteroventral seta, except black dorsal setulae at apical half; hind femur with black dorsal and pale ventral setulae and 1 apical anterodorsal and 1 posteroventral seta; all tibiae with 1–3 anterodorsals and 1–3 posterodorsal setae and apical ring of setae; all tarsi with 2 long ventral setae at apex of tarsomeres 1–4, respectively; tarsomere 1 grey and almost same or slightly shorter than total length of tarsomeres 2–5; tarsomeres 2­–5 basally yellow and apically grey; fore tarsomere 4 with anterior projection and 2 setae at apex; fore tarsomere 5 anteriorly crooked and gradually widened towards apex and with anterior projection at base; mid tarsomeres 4 and 5 gradually broadened towards apex; mid tarsomeres 5 cone-shaped; hind tarsomeres 4 and 5 slightly broadened towards apex, but less broad than mid tarsomeres 4 and 5; all claws long, approximately twice longer than pulvillus; basal half of fore anterior claw broad, almost equal to width of pulvillus; all empodium pale, narrow feather shaped and curved upwards; pulvillus greyish brown (Fig. 1F-H); length of tibia and tarsus of fore leg (mm), 1.59 : 0.65 : 0.16 : 0.13 : 0.8 : 0.31; mid leg, 1.81 : 1.15 : 0.29 : 0.27 : 0.19 : 0.34; hind leg, 2.31 : 1.34 : 0.36 : 0.32 : 0.26 : 0.36.

**Wing** simple and hyaline with dark brown veins; C ending at M_1_; Sc combined at half of R_1_; R_2+3_ and R_4+5_ slightly diverging towards wing tip; R_4+5_ and M_1_ divergent before dm-m, then slightly convergent towards wing tip; r-m crossed at branching point of R_2+3_ and R_4+5_; M_4_ fold-like at wing tip; dm-m straight; CuA+CuP fold-like and ending before reaching wing tip; alula absent (Fig. 1D); calypter pale yellow with pale setulae; halter pale yellow with brown base.

**Figure 2.**
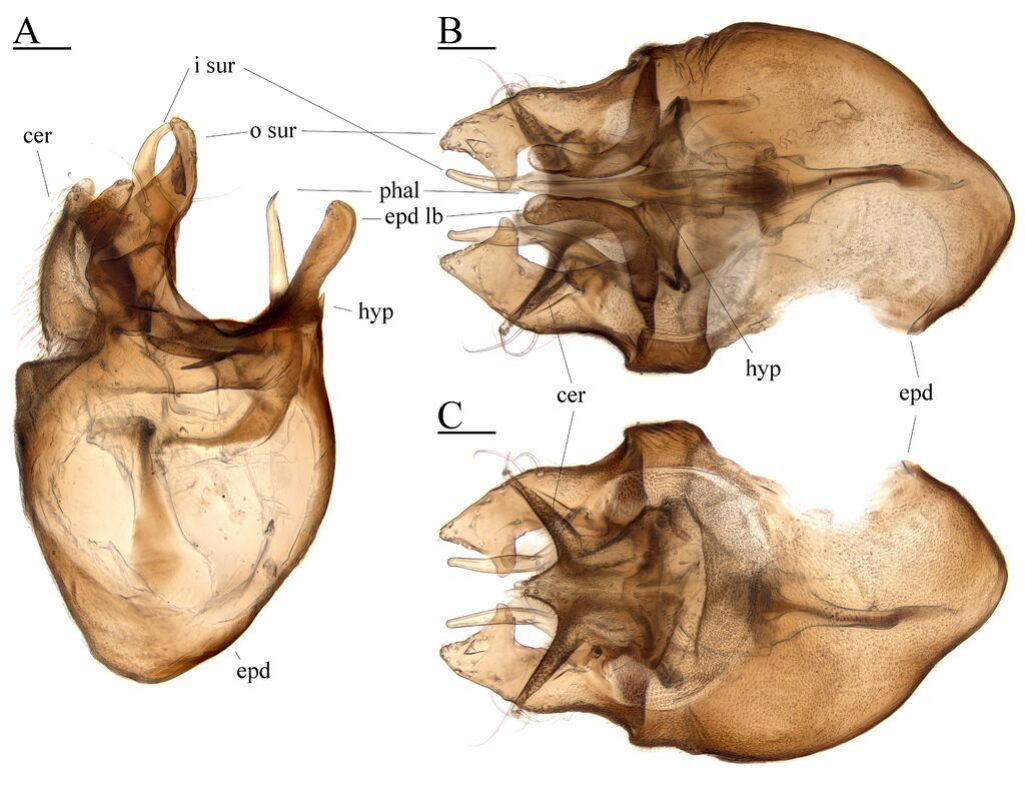
*Hypocharassuscavitarsus* sp. n. male. **A** genitalia, lateral view; **B** ditto, ventral view; **C** ditto, dorsal view. Scale bars: A-C = 0.1 mm. Abbreviations: cer = cercus; epd = epandrium; hyp = hypandrium; i sur = inner surstylus; o sur = outer surstylus; phal =phallus.

**Abdomen** metallic bluish green with whitish pruinosity; tergum with black setulae, except lateral pale setulae; sternum with pale setulae; sternite 1 membranous, except lateral small sclerotised apex; sternite 4 with small spikes at posterior median apex; sternite 5 medially concave and membranous (Fig. 1E); epandrium dorsally broad, slightly broader than height; hypandrium fused to epandrium; epandrial lobe bacilli form, approximately 2.5 times as long as width; inner surstylus dorsally crooked bacilli form; outer surstylus dorsally broad with inner projection; phallus narrow and slightly crooked to venter at apex; cerci fused together and forming a trident shape (Fig. 2).

Length: body without antenna 5.1–5.3 mm, antenna 0.9–1.0 mm and wing 4.7–5.5 mm.

***Female*** (Fig. 1B)

Almost identical to male, except for the following characteristics: all tarsomeres 4 and 5 only minutely broadened towards apex, fore and mid tarsus without any specific modification like male; sternite 4 without small spikes at posterior median apex; sternite 5 not medially concave and membranous.

Length: body without antenna 5.6–6.1 mm, antenna 0.9–1.0 mm and wing 6.2–6.5 mm.

#### Etymology

The species name is derived from a Latin word that translates to concave (cavus) tarsomere 5 of fore tarsus.

#### Distribution

Korea (Chungcheongnam-do, Jeollanam-do).

## Identification Keys

### Key to *Hypocharassus* species worldwide

**Table:** 

1	Scape with dorsal setae	2
–	Scape without dorsal setae	3
2	Ventral projection of postpedicel long, approximately 0.5 times as long as dorsal projection (eastern USA)	*H.pruinosus* (Wheeler, 1898)
–	Ventral projection of postpedicel short, approximately 0.1 times as long as dorsal projection (south-eastern USA)	*H.gladiator* Mik, 1879
3	Large species, approximately 13 mm (southern China)	*H.sinensis* Yang, 1998
–	Small species, approximately 6~7 mm	4
4	Male fore tarsus simply widened towards apex, without anterior projection at base (Fig. 3); halter brown (Taiwan)	*H.farinosus* Becker, 1922
–	Male fore tarsus widened towards apex, with anterior projection at base (Fig. 1F); halter yellow (Korea)	*H.cavitarsus* sp. n.

Based on Wheeler (1898) and Yang (1998)

## Discussion

**Figure 3.**
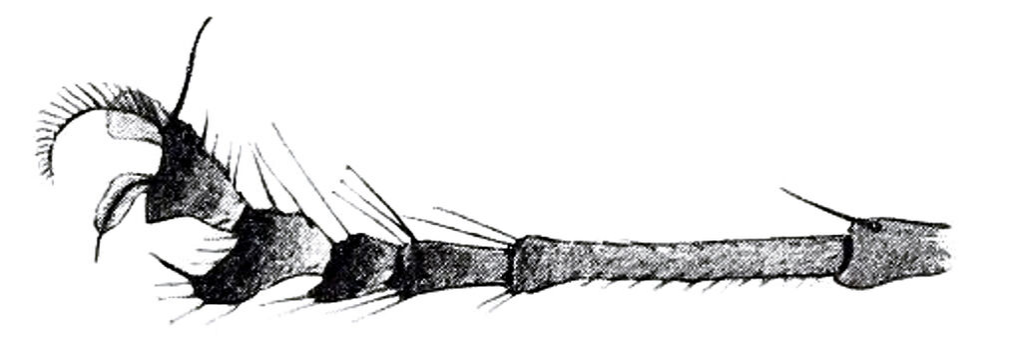
*Hypocharassusfarinosus* Becker, 1922 male fore tarsus (Becker 1922- (https://www.biodiversitylibrary.org/page/53707851).

This new species and Oriental species can be distinguished easily from two Nearctic species by the absence of scape setae. While, compared to new species and Oriental species, they have relatively similar morphological features. However, each can be identified by the following characteristics. *Hypocharassussinensis* Yang, 1998 which is identified by the female type specimen, can be separated from the new species by five pairs of scutellar setae and two times larger body size (approximately 13 mm). *H.farinosus* Becker, 1922 also can be distinguished from the new species by the brown halter and calypter, absence of anterior projection at base of fore tarsomere 5 and absence of basally broad fore anterior claw (Fig. 3).

## Supplementary Material

XML Treatment for Hypocharassus cavitarsus

## References

[B7657435] Becker T. (1922). Dipterologische studien; Dolichopodidae der Indo-Australischen region. Capita Zoologica.

[B7659949] Coquillett D. W. (1910). The type-species of the North American genera of Diptera. Proceedings of the United States National Museum.

[B7657470] Mik J. (1878). Dipterologische Beiträge. Verhandlungen der Kaiserlich-Königlichen Zoologisch-Botanischen Gesellschaft in Wien.

[B7657479] Smith M. (1952). Immature stages of the marine fly, *Hypocharassuspruinosus* Wh., with a review of the biology of immature Dolichopodidae. American Midland Naturalist.

[B7657488] Wheeler W. M. (1898). A new genus of Dolichopodidae from Florida. Zoölogical Bulletin.

[B7657601] Wheeler W. M. (1900). On the genus *Hypacharassus* Mik. Entomological news, and proceedings of the Entomological Section of the Academy of Natural Sciences of Philadelphia.

[B7657497] Yang D. (1998). New and little known species of Dolichopodidae from China (I). Bulletin de l’Institute Royal des Sciences Naturelles de Belgique, Entomologie.

